# A novel porous mechanical framework for modelling the interaction between coronary perfusion and myocardial mechanics

**DOI:** 10.1016/j.jbiomech.2011.11.026

**Published:** 2012-03-15

**Authors:** A.N. Cookson, J. Lee, C. Michler, R. Chabiniok, E. Hyde, D.A. Nordsletten, M. Sinclair, M. Siebes, N.P. Smith

**Affiliations:** a Imaging Sciences & Biomedical Engineering Division, St Thomas' Hospital, King's College London, SE1 7EH, UK; b Department of Computer Science, Oxford OX1 3QD, UK; c Biomedical Engineering and Physics, Academic Medical Center, University of Amsterdam, 1105 AZ, The Netherlands

**Keywords:** Incompressible poroelastic media, Multi-compartment, Coronary perfusion, Myocardial mechanics, Finite element method

## Abstract

The strong coupling between the flow in coronary vessels and the mechanical deformation of the myocardial tissue is a central feature of cardiac physiology and must therefore be accounted for by models of coronary perfusion. Currently available geometrically explicit vascular models fail to capture this interaction satisfactorily, are numerically intractable for whole organ simulations, and are difficult to parameterise in human contexts. To address these issues, in this study, a finite element formulation of an incompressible, poroelastic model of myocardial perfusion is presented. Using high-resolution ex vivo imaging data of the coronary tree, the permeability tensors of the porous medium were mapped onto a mesh of the corresponding left ventricular geometry. The resultant tensor field characterises not only the distinct perfusion regions that are observed in experimental data, but also the wide range of vascular length scales present in the coronary tree, through a multi-compartment porous model. Finite deformation mechanics are solved using a macroscopic constitutive law that defines the coupling between the fluid and solid phases of the porous medium. Results are presented for the perfusion of the left ventricle under passive inflation that show wall-stiffening associated with perfusion, and that show the significance of a non-hierarchical multi-compartment model within a particular perfusion territory.

## Nomenclature

β: coupling coefficient between fluid compartments
F: deformation gradient tensor
K: permeability tensor of porous medium
M: Lagrangian Darcy flow vector
S: second Piola–Kirchhoff stress tensor
$w_{i}$: Eulerian relative flow vector
X: reference coordinates
x: deformed coordinates
λ: Lagrange multiplier that enforces volume constraint
$V_{f}$: velocity of fluid
$V_{s}$: velocity of solid
$\mu_{f}$: dynamic viscosity of the fluid
$\phi_{f}$: porosity of fluid phase
$\phi_{s}$: porosity of solid phase
$\rho_{f}$: density of fluid phase
*J*: determinant of F
*m*: fluid mass increase
*p*: pressure
*q*_*i*_: volumetric source term

## 1 Introduction

The perfusion of the heart is inherently coupled to its mechanical state. This coupling is perhaps most clearly evidenced in the large epicardial coronary vessels within which flow is impeded and even reversed during contraction. However, this epicardial phenomenon is fundamentally produced by compression of vessels within the myocardium which, in turn, increases their resistance to flow resulting in a reduced/reversed proximal pressure gradient. The interplay between the dynamics of vessel compression with resistance and pressure gradients has motivated the development of a number of modelling frameworks. Early examples include the vascular waterfall model first proposed by Downey and Kirk (1975), which was further developed within the seminal intramyocardial pump (Spaan et al., 1981), variable elastance (Krams et al., 1990) and coronary models (see Westerhof et al., 2006 for a detailed review).

Advances in modelling techniques and high performance computing have enabled the coupling between coronary flow and myocardial deformation to be simulated within spatially discrete frameworks (Huo et al., 2009; Smith, 2004; Smith and Kassab, 2001). These approaches, combined with recent developments in high resolution imaging, now provide frameworks (Kaneko et al., 2011; van den Wijngaard et al., 2011; Lee et al., 2007) within which to analyse coronary flow on an explicit computational representation of vascular networks.

However, despite the progress of the field and the insights which have already been gained, detailed coronary anatomical information is unlikely to be available in clinical contexts where perfusion is typically assessed by observing the passage of an imaging contrast agent. Furthermore, the majority of existing models do not account for the effect of coronary blood pressure on myocardial tissue models, despite its influence being likely to be physiologically significant (Dijkman et al., 1998).

Recent work has addressed this issue through the development of poro-elastic mechanical models of myocardial tissue (Huyghe et al., 1992; May-Newman and McCulloch, 1998; Chapelle et al., 2010), which have progressively incorporated additional complexities associated with representing perfused myocardium. The model of Huyghe et al. (1992) assumed quasi-static fluid flow and a quasi-linear viscoelastic constitutive law. The approach taken by May-Newman and McCulloch (1998) was to assume an idealised representation of the vascular embedding within the tissue, and perform a homogenisation procedure to obtain a non-linear constitutive relation for the solid mechanical behaviour. More recently, the model presented by Chapelle et al. (2010) included unsteady fluid flow, and a non-linear hyperelastic material, which was assumed to be nearly incompressible.

While each of these studies has made a significant contribution to the development and application of poro-mechanical frameworks for capturing coronary perfusion, a number of limitations persist. Specifically all of these models were applied to idealised, axisymmetric representations of the left ventricle, and all three employed a single fluid phase/porous compartment to represent the coronary vasculature. This approach limits the ability of the model to directly parameterise permeability tensors of the porous medium from vascular data and thus exploit information derived from detailed coronary anatomies.

In this paper we aim to address these issues through the development and application of a multi-compartment coupled porous-mechanical model of the cardiac left ventricle, providing a modelling framework for the future integration of both detailed structural and clinical imaging data.

## 2 Mathematical formulation of a multi-compartment poroelastic model

### 2.1 Multi-compartment porous medium

Consider a simple saturated porous medium consisting of a fluid and a solid phase. Its total mass can be defined as

(1) $$m_{\mathrm{total}}=\int_{\Omega }(\rho_{f}\phi_{f}+\rho_{s}\phi_{s})\;d\Omega ,$$

where ϕ and ρ refer to the volume fractions occupied by, and density of, each of the phases. Note that the integral bound is the total domain Ω, such that

(2) $$\phi_{f}+\phi_{s}=1.$$

Note however that unless the solid is rigid, the bulk porous medium will be compressible, regardless of the compressibility of the individual constituents, as the pore space can undergo volume changes due to net fluid inflow.

In the vascularised tissue such as the cardiac tissue, the vessel flow direction displays significant heterogeneity even in close spatial proximity, and the vessels themselves span multiple scales (radius μm–mm, length μm–cm). Due to these reasons it is more useful to treat each of the functional groups of the vascular tree as its own pore space, or compartment, which is connected to other compartments.

Thus the single fluid phase generalised to *N* compartments, all of which occupy a fraction of the total volume such that

(3) $$\overset{N}{\underset{i}{\sum }}\phi_{f,i}+\phi_{s}=1.$$

This formulation offers two advantages. Firstly, it preserves the hierarchy of flow between nearby vessels of disparate length scales, which a single-compartment formulation would otherwise smear out. Secondly, it allows the parameters such as permeability to be represented distinctly for each compartment, thus allowing greater accuracy in material characterization.

A similar concept was previously introduced by Vankan et al. (1997) in a 2D model of perfusion in rat calf muscle, in which the flow between compartments was restricted to a strictly hierarchical manner. In the framework presented below, the cross-compartment connections between all levels, which are evident from studies of vascular morphology (Kassab et al., 1993), are captured using a generalization of the previously applied double porosity network concept (Coussy, 2004).

### 2.2 Multi-compartment poroelastic flow equations

The evolution equations for both the fluid and solid phases are described here, formulated in a more naturally suited Lagrangian form. The equations governing flow through deformable porous media are given by Coussy (2004). Following standard conventions, reference and deformed coordinates are denoted by X and x, respectively, y=x−X is the displacement of the skeleton, and

(4) $$F=\frac{\partial x}{\partial X}\;\mathrm{and}\;J=\det\;F$$

denote the deformation gradient tensor and its associated Jacobian, respectively.

The equations of Darcy flow for compartment *i* in a system of *N* compartments, where i,k=1,…,N denote the compartment indices, are

(5a) $$\frac{F\cdot M_{i}}{\rho_{f}}=-JK_{i}F^{-T}\nabla_{X}p_{i},$$

(5b) $$\frac{d^{s}m_{i}}{\mathrm{dt}}+\nabla_{X}\cdot M_{i}=\overset{N}{\underset{k=1}{\sum }}-J\beta_{i,k}(p_{i}-p_{k})+\rho_{f}q_{i},$$

variable $M_{i}$ is the Lagrangian Darcy flow vector, related to the Eulerian relative flow vector of fluid mass by

(6a) $$M_{i}=JF^{-1}\cdot w_{i}$$

with

(6b) $$w_{i}=\rho_{f}\phi_{f,i}(V_{i}^{f}-V^{s}),$$

where $V_{i}^{f}$ and $V^{s}$ denote the velocity of the fluid and of the skeleton particle, respectively.

The fluid density is $\rho_{f}$, pressure is *p*_*i*_, $K_{i}$ is the permeability tensor of the porous medium, and *q*_*i*_ is a volumetric source term. The pressure is determined using a constitutive law, the details of which are given in Eqs. (10)–(13).

In the equation of mass conservation, Eq. (5b), the operator $d^{s}m_{i}/\mathrm{dt}$ is the particle derivative with respect to the skeleton, such that

(7) $$\frac{d^{s}m_{i}}{\mathrm{dt}}=\frac{\partial m_{i}}{\partial t}+(\nabla_{x}m_{i})\cdot V^{s},$$

where *m*_*i*_ is the fluid mass increase of compartment *i*, defined per unit volume of the reference configuration. The coefficient $\beta_{i,k}$ describes the flow coupling between compartments and is defined as (Coussy, 2004)

(8) $$\beta_{i,k}=\frac{\nu_{i,k}\rho_{f}}{\mu_{f}},$$

where $\nu_{i,k}$ is a dimensionless coefficient, which describes the permeability between two compartments and $\mu_{f}$ is the dynamic viscosity of the fluid. It is assumed that the communication between compartments occurs solely as a mass exchange proportional to the pressure difference. Momentum transfer associated with this mass exchange is assumed to be negligible. To attain a conservative exchange between compartments *i* and *k* it follows that $\beta_{i,k}$ must be symmetric, i.e. the mass drained from (fed into) compartment *i* equals the mass fed into (drained from) compartment *k*, and $\beta_{i,i}=0$.

### 2.3 Finite-deformation elasticity

We model the kinematics of the skeleton by finite-deformation elasticity to allow for large strains. The skeleton kinematics are governed by the linear momentum balance

(9a) $$\nabla_{X}\cdot (\mathrm{FS})=0,$$

where S is the second Piola–Kirchhoff stress tensor. Eq. (9a) is solved subject to the volume constraint

(9b) $$J=1+\frac{\sum_{i}^{N}m_{i}}{\rho_{f}},$$

which is enforced using a Lagrange multiplier, denoted by λ, where *m*_*i*_ is the fluid mass increase determined from Eq. (5b). Incompressibility of the solid matrix is implied by Eq. (9b), that is, any volume increase (decrease) can occur only by pore dilatation (contraction) due to an increase (decrease) of pore fluid mass.

The constitutive law is given by Eq. (10), which is an isotropic exponential-form material law, modified to account for the effect of fluid mass increase on the strain energy through the use of the $m_{i}/\rho_{f}$ terms. Compared to the standard finite elasticity constitutive laws this law additionally governs the pore pressure development, as well as the skeletal stress, and characterises the coupling between solid and fluid media. For multi-compartment models, *Q* ^*i*^_*j*_ is allowed to differ for each compartment, to capture the differing vessel compliances that exist.

(10) $$\Psi^{s}=a\left[\exp\left(D_{1}\left({\overset{¯}{I}}_{1}\left(1+\overset{N}{\underset{i}{\sum }}Q_{1}^{i}\frac{m_{i}}{\rho_{f}}\right)-3\right)+D_{2}\left({\overset{¯}{I}}_{2}\left(1+\overset{N}{\underset{i}{\sum }}Q_{2}^{i}\frac{m_{i}}{\rho_{f}}\right)-3\right)\right)\right)+\left(\left(D_{3}\left({\left(J-1\right)}^{2}+\overset{N}{\underset{i}{\sum }}Q_{3}^{i}{\left(\frac{m_{i}}{\rho_{f}}\right)}^{2}\right)\right)-1\right],$$

where *I*¯_1_ and *I*¯_2_ denote the modified invariants of the right Cauchy–Green deformation tensor $C=F^{T}F$, defined as *I*¯_1_=*J* ^−2/3^ *I*_1_ and *I*¯_2_=*J* ^−4/3^ *I*_2_. The terms *a*, *D*_1_, *D*_2_, *D*_3_, $Q_{1}^{i}$, $Q_{2}^{i}$ and $Q_{3}^{i}$ are material parameters.

The constitutive law is then subjected to the constraint Eq. (9b) to form

(11) $$\Psi_{\mathrm{cons}}^{s}=\Psi^{s}+\lambda \left(J-1-\overset{N}{\underset{i}{\sum }}\frac{m_{i}}{\rho_{f}}\right).$$

The second Piola–Kirchhoff stress tensor π is then defined as (see Bonet and Wood, 2008, Chapter 5.5.1)

(12) $$\pi =\frac{\partial \Psi^{s}}{\partial E}+\lambda JC^{-1},$$

while the relationship between the compartmental fluid pressure, *p*_*i*_, and the constitutive law is given by

(13) $$p_{i}=\frac{\partial \Psi^{s}}{\partial (J\phi_{f,i})}-\lambda .$$

## 3 Computational methods

### 3.1 Partitioned solution strategy of the coupled equations

A partitioned scheme is chosen to solve the coupled solid–fluid equations, with fixed point sub-iterations between solid and fluid solution steps used to obtain a converged solution at each time step.

It is possible to eliminate $M_{i}$, reducing the number of equations to three, by substituting $M_{i}$ defined in Eq. (5a) into Eq. (5b) to yield

(14) $$\frac{d^{s}m_{i}}{\mathrm{dt}}=-\nabla_{X}\cdot (-\rho_{f}JF^{-1}K_{i}F^{-T}\nabla_{X}p_{i})+\rho_{f}q_{i}+\overset{N}{\underset{k=1}{\sum }}-J\beta_{i,k}(p_{i}-p_{k}).$$

Although the flow vector $M_{i}$ is a quantity of interest, it is not necessary for the coupling with the solid, which occurs through mass increase, *m*_*i*_ and Lagrange multiplier, λ. Therefore, during the sub-iterations between solid and fluid solution steps, a saving in computational cost can be obtained by solving only Eq. (14), rather than both Eqs. (5a) and (5b). Once the converged solution is reached, the fluid velocity is then determined using Eq. (5a).

### 3.2 Finite element formulation

The discretization of the governing equations (9) and (14) is performed using a standard Galerkin finite-element discretization in space with Lagrange basis functions. In particular, displacement y is discretized using a quadratic basis, while λ, *p*_*i*_, $M_{i}$ and mass increase *m*_*i*_ are represented by a linear basis. A backward Euler method is used to perform the time integration of Eq. (14).

### 3.3 Model parameterisation

#### 3.3.1 Poroelastic constitutive law

**Fig. 1:**
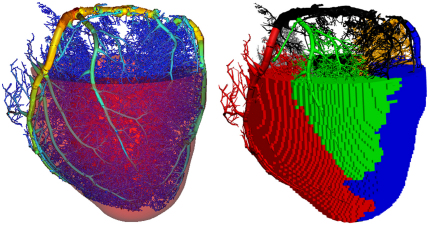
The vascular tree for vessels with a radius in the range 0.031–1.9 mm, which was reconstructed from cryomicrotome imaging data. This is embedded within the surface representation of the left ventricle geometry, with the vessels coloured by radius (*left*). The main subtrees of the vasculature are then identified from this tree, and the tissue associated with each subnetwork determined by a discrete distance metric (*right*).

**Fig. 2:**
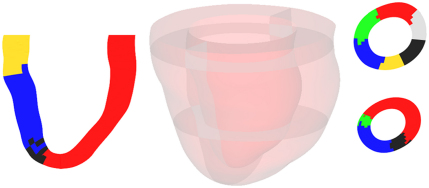
Various cross-sections of the left ventricle (LV) model showing the results of the regional partition for a chosen set of six subnetworks that perfuse the LV. Each colour is a distinct, contiguous region of tissue associated with a particular subnetwork. The central figure indicates the position of the cross-sections relative to the LV model, the left-hand column is a long-axis cross-section while the right-hand column shows short-axis cross-sections.

**Fig. 3:**
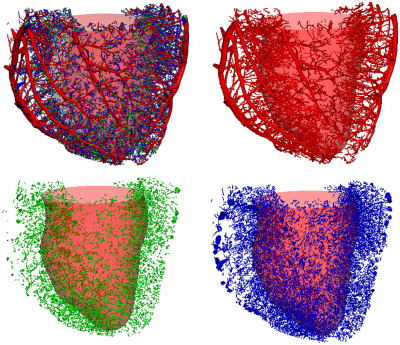
The full vascular tree (*upper left*), vessels belonging to compartment one (*upper right*), vessels belonging to compartment two (*lower left*), and vessels belonging to compartment three (*lower right*).

High resolution cryomicrotome imaging data of myocardial vasculature strongly suggests that the myocardium is partitioned into regional perfusion zones, and that these regions are perfused by distinct arterial subnetworks (Spaan et al., 2005). To capture this within the model every such region is associated to its unique subnetwork using the morphology to determine regional boundaries of zero normal flux, and to derive a region-specific permeability tensor. This regional partitioning for a selection of six subnetworks that supply the left ventricle (LV) is shown in Figs. 1 and 2 . Such inclusion of accurate anatomical data is an essential step towards reproducing physiologically based perfusion of the myocardium. The division of the vascular tree into three compartments is illustrated in Fig. 3 .

A Principal Components Analysis (PCA) technique was employed in order to base this spatially averaged parameter field derivation on the vascular data. Specifically, the regional subnetwork was discretized into a number of smaller segments. The unit orientation vector of each segment from compartment *i* constitutes a single datum in the PCA, which was then weighted by its segment conductance. This segment property was chosen assuming parabolic flow through an idealised cylindrical segment, with the product of the conductance and the applied pressure gradient yielding the flux through the segment. Once constructed, the generated data set for that regional subnetwork was then subjected to PCA, yielding a positive, semi-definite permeability tensor, $K_{i}$. The proportion of fluid volume to total material volume within a region defines the regional porosity.

The constitutive parameters were obtained through manual tuning, which yielded the following values *a*=1.0, $D_{1}=2.0$, $D_{2}=0.2$, $D_{3}=2.0$ and $Q_{1}^{i}=1.0$, $Q_{2}^{i}=0.5$ and $Q_{3}^{i}=1.0$.

## 4 Results

**Fig. 4:**
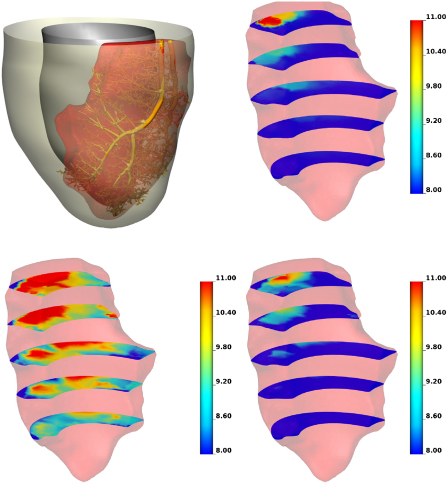
The discrete network embedded within its distinct perfusion territory on which a comparison of a Poiseuille flow model and a continuum model based on Darcy's law is made (*upper left*). Volume-averaged pressure, plotted on five axial slices, from the Poiseuille flow model (*upper right*) is matched well by the static Darcy model (*lower right*). Comparison with the strictly hierarchical model (*lower left*) shows that removing the connections between non-neighbouring compartments results in an over-estimation of pressure.

In order to test the accuracy of the continuum porous model in representing the discrete blood velocity, a Poiseuille flow computation was performed on the discrete vessel network. This discrete solution was then volume averaged to allow comparison with the continuum model. The pressure in the Darcy model was obtained by a volume-weighted average of all the compartmental pressures. The results of this comparison are shown in Fig. 4 , and reveal a good agreement in the magnitude and spatial variation of pressure.

As discussed in the Introduction and outlined in Section 2 the multi-compartment formulation allows for the possibility of connections between non-neighbouring compartments, rather than enforcing a strict hierarchy of flow, whereby fluid must pass through compartment two if it is to flow from compartment one to three. The provision for this was motivated by the properties of the vascular tree data. In Fig. 4a comparison of the two models is presented, which clearly illustrates the higher pressure values that occur in the strictly hierarchical model. This is caused by the lower overall permeability of the hierarchical system, than in the case where the non-hierarchical connections are included.

To study the fluid–solid coupling behaviours of the model we simulated passive inflation of a full left ventricle (Lamata, 2011), with a diastolic cavity pressure ramp. In order to demonstrate the wall stiffening behaviour caused by fluid mass increase, a fluid source, corresponding to a linear ramp up to an 8% overall wall volume increase, has been simultaneously applied with the cavity inflation in one of the simulations. To isolate the mechanism of the garden-hose effect, spatially uniform source strengths and permeabilities were employed, thereby avoiding the potentially confounding effects of detailed parameterisation.

**Fig. 5:**
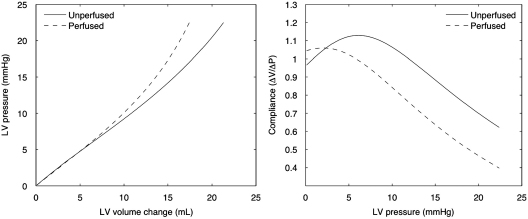
Plot of LV cavity volume for various cavity pressures, for both perfused and unperfused states (*left*). The smaller increase in cavity volume for the perfused state indicates stiffening of the ventricle wall, which is quantified in the plot of compliance versus LV cavity pressure (*right*).

Fig. 5 shows that as the fluid content of the tissue increases, the LV progressively stiffens. Estimations of instantaneous compliance show a settling trend to a relatively constant difference between the perfused and unperfused cases, consistent with that reported in May-Newman et al. (1994). However, the initial trends in compliances show that at very low states of perfusion, the tissue stiffening caused by the mass inflow can be overcome by the deformation that the fluid mass itself induces. With the current isotropic constitutive law, this strain occurs largely in the longitudinal direction, as opposed to the radial direction as reported in the experimental study, highlighting the need for further work required in cardiac poroelastic constitutive laws.

## 5 Conclusions

The presented results show that the proposed modelling framework of a multi-compartment poroelastic medium has the potential to serve for the investigation of coronary perfusion phenomena. In particular, the multi-compartment porous medium was shown to provide a good approximation to a discrete model of the coronary tree. In addition, the model reproduced the expected wall stiffening due to perfusion increasing the fluid mass content in the wall.

One of the difficulties found in these initial modelling studies was the application of realistic fluid sources and boundary conditions on the fluid model. The choice of distributed sources over an input velocity condition was justified by the morphological characteristics of the vascular network, in which the progressive branching to smaller vessel segments can be regarded as entering a fluid compartment over a distributed volume. Similarly, the sinks correspond to smaller vessel segments which are further distributed in space and carry the flow to the next compartment. Nevertheless, further work is underway to move beyond the simple specified volume rate conditions, by coupling the 1D explicit vascular model to the 3D poroelastic model, to address this challenge.

To validate the modelling approach, high-resolution perfusion data obtained under controlled experimental conditions are required. Proof-of-concept work to obtain such data has been conducted (Schuster et al., 2010) and comparison with the experiments is an ongoing focus in our group.

Future work will focus on further development of the constitutive relations as well as detailed parameterisation from animal and human data, and clinical validation using magnetic resonance perfusion imaging.
